# GalaxyHeteromer: protein heterodimer structure prediction by template-based and *ab initio* docking

**DOI:** 10.1093/nar/gkab422

**Published:** 2021-05-28

**Authors:** Taeyong Park, Jonghun Won, Minkyung Baek, Chaok Seok

**Affiliations:** Department of Chemistry, Seoul National University, Seoul 08826, Republic of Korea; Department of Chemistry, Seoul National University, Seoul 08826, Republic of Korea; Galux Inc., Seoul 08826, Republic of Korea; Department of Chemistry, Seoul National University, Seoul 08826, Republic of Korea; Department of Chemistry, Seoul National University, Seoul 08826, Republic of Korea; Galux Inc., Seoul 08826, Republic of Korea

## Abstract

Protein–protein interactions play crucial roles in diverse biological processes, including various disease progressions. Atomistic structural details of protein–protein interactions may provide important information that can facilitate the design of therapeutic agents. GalaxyHeteromer is a freely available automatic web server (http://galaxy.seoklab.org/heteromer) that predicts protein heterodimer complex structures from two subunit protein sequences or structures. When subunit structures are unavailable, they are predicted by template- or distance-prediction-based modelling methods. Heterodimer complex structures can be predicted by both template-based and *ab initio* docking, depending on the template's availability. Structural templates are detected from the protein structure database based on both the sequence and structure similarities. The templates for heterodimers may be selected from monomer and homo-oligomer structures, as well as from hetero-oligomers, owing to the evolutionary relationships of heterodimers with domains of monomers or subunits of homo-oligomers. In addition, the server employs one of the best *ab initio* docking methods when heterodimer templates are unavailable. The multiple heterodimer structure models and the associated scores, which are provided by the web server, may be further examined by user to test or develop functional hypotheses or to design new functional molecules.

## Graphical Abstract

**Graphical Abstract:**
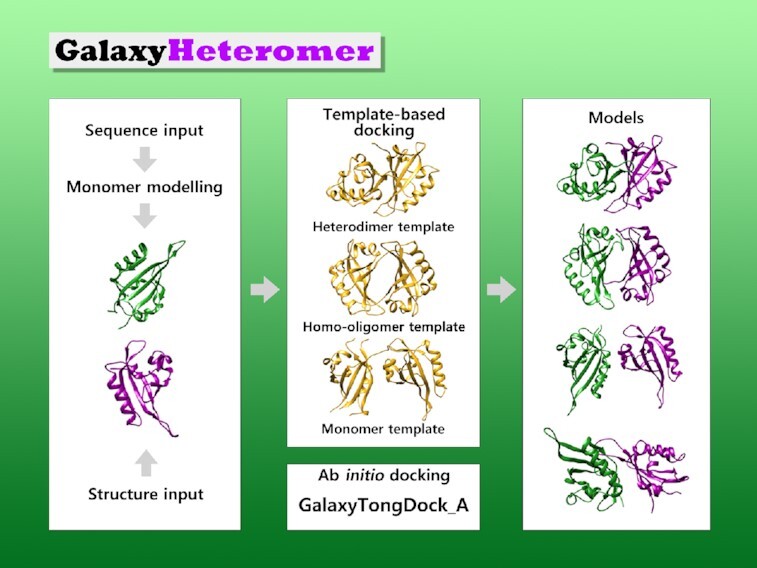
GalaxyHeteromer is a freely available automatic web server (http://galaxy.seoklab.org/heteromer) that predicts protein heterodimer complex structures from two subunit protein sequences or structures. When subunit structures are unavailable, they are predicted by template- or distance-prediction-based modelling methods. Heterodimer complex structures can be predicted by both template-based and ab initio docking, depending on the template’s availability.

## INTRODUCTION

Protein–protein interactions (PPIs) play key roles in a wide range of biological processes, ranging from development and ageing to various disease progressions (1–3). Therefore, understanding the atomistic detail of PPIs is a crucial prerequisite for identifying therapeutic molecules that inhibit PPIs. Computational methods for protein–protein complex structure prediction have been used as a valuable tool for the atomic-level understanding of PPIs due to the limited number of available protein–protein complex structures obtained experimentally, especially for transient or weak protein–protein complexes (4–7).

Protein–protein complex structures are currently predicted using template-based or *ab initio* docking (8–14), depending on the availability of structural templates for the target complex in the structure database. Structural templates for a protein–protein complex can be detected by exploiting sequence or structure similarities of consisting subunit proteins to proteins in the database. When such similarity-based approaches are not reliable due to the lack of available structural templates, *ab initio* docking, which is based on the physical principles of protein binding, is used. *Ab initio* docking identifies the most stable binding pose in the conformational space of protein–protein complexes by conformational sampling and stability evaluation. The performances of complex structure prediction methods are continuously improving in both template-based and *ab initio* docking, according to the results of recent community-wide prediction experiments CASP (15) and CAPRI (16,17).

Herein, we introduce a new web server, GalaxyHeteromer, that predicts heterodimer protein–protein complex structure from amino acid sequences or structures of two different subunit proteins composing the heterodimer. Both template-based and *ab initio* docking are employed by automatically detecting the template's availability. GalaxyHeteromer utilizes a modern structure prediction method, which employs inter-residue distance prediction by exploring the coevolution relationships among the homologous sequences via deep learning, for subunit structure prediction (18). This advanced subunit structure prediction can result in more accurate prediction of the complex structure. The server also employs an extensive structure database for searching the template, encompassing monomers, homo-oligomers, and hetero-oligomers to explore possible evolutionary relationships of subunit proteins to domains of monomers or subunits of homo- and hetero-oligomers. For *ab initio* docking, the server uses an effective docking method, known as GalaxyTongDock_A, which was developed through a systematic search of energy parameters for pose stability evaluation (14).

## THE GALAXYHETEROMER METHOD

### Overall pipeline

**Figure 1.**
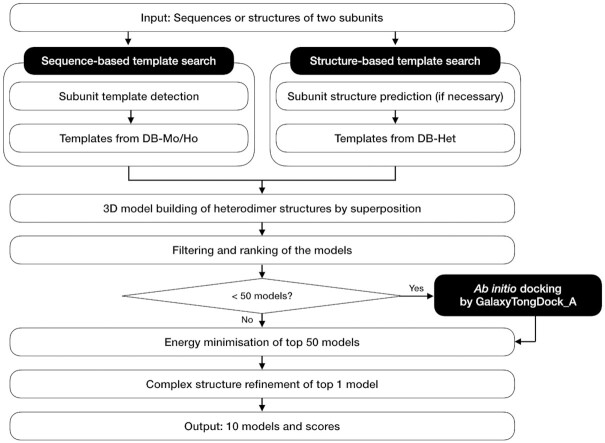
GalaxyHeteromer pipeline for heterodimer protein complex structure prediction.

The prediction pipeline of the GalaxyHeteromer server for predicting heterodimer structure is shown in Figure 1. In GalaxyHeteromer, template-based docking is performed by detecting templates for heterodimer structure building based on subunit sequence similarities (sequence-based template search) and subunit structure similarities (structure-based template search), as described in detail below. If subunit structures are not provided as input, they are predicted from subunit sequences using a recently developed protein structure prediction method explained below. Then, 3D models for heterodimer structures are generated by superposing the subunit structures on the template structures. The models are filtered based on physical criteria, such as steric clashes, inter-subunit contacts, and interface area. After removing redundancy (of TM-score (19) > 0.8) among the heterodimer models, the models are ranked according to a template score, which consists of subunit and interface structure similarities measured in TM-score to the template structures. If <50 models are left, *ab initio* docking is performed using GalaxyTongDock_A (14) to generate more models, so that a total of 50 models can be obtained. After energy minimization, the best scoring model is further refined by re-modelling interfacial loop structures which were detected as inaccurate by the loop modelling method GalaxyLoop (20), and relaxed by repetitive side chain perturbations and molecular dynamics simulations using the complex structure refinement method GalaxyRefineComplex (21).

### Subunit structure prediction

When only subunit sequences are provided as input, subunit structures are predicted from sequences by the protein structure method of GALAXY group who participated in CASP14 (2020) as Seok-server. This method selects a model through a random forest classifier from the models that were predicted by template-based structure prediction method, GalaxyTBM (22), and from those that were predicted by a distance-prediction-based structure prediction method, GalaxyDBM (unpublished). The accuracy of this method is comparable to that of AlphaFold (18) on CASP13 targets (https://predictioncenter.org/casp13/), in terms of the CASP measure GDT-TS (23) (average GDT-TS = 62.3, whereas that of AlphaFold is 62.9).

GalaxyDBM predicts the probability distributions over distances between C-beta atoms (C-alpha for GLY) of different residues using a deep residual convolutional neural network, which is based on MSA-based features, including sequence profile and raw coevolutionary coupling features from CCMPred (24), following AlphaFold (18). Thereafter, 3D backbone structures are predicted by the global optimization method, which is known as conformational space annealing (25), maximizing the likelihood of probability distributions and satisfying local stereochemistry controlled by GALAXY energy function (20,26). The predicted structures are then refined by GalaxyRefine (27) for optimizing side chain conformations.

### Sequence- and structure-based template search

Sequence-based template search is performed on the database of monomer and homo-oligomer proteins, named DB-Mo/Ho, as shown in Figure 1. DB-Mo/Ho is the protein structure database of monomers and homo-oligomers, which has a maximum mutual sequence identity of 70%. Subunit templates are first detected by HHsearch (28). Proteins in DB-Mo/Ho with high sequence similarity (in terms of GalaxyTBM template score (22) within top 200) and high structure similarity (TM-score > 0.4) to both subunits in different parts of the same protein (e.g. different domains of a monomer or different subunits of a homo-oligomer) are selected as templates for building the heterodimer structure. Proteins with interface structure similarity of less than TM-score < 0.4 are discarded for homo-oligomer templates.

Structure-based template search is performed on the database of heterodimers, named DB-Het, as shown in Figure 1. DB-Het was prepared by collecting non-redundant heterodimer structures from protein complex structures of resolution better than 4.0 Å in PDB, which consist of more than two distinct proteins. DB-Het comprised 45 267 heterodimers as of March 2021, and it will be updated regularly. Structure-based templates are detected by finding heterodimers with high subunit structure similarities (TM-score > 0.4) to both subunits. Proteins with interface structure similarity less than TM-score < 0.4 are discarded.

### Performance of GalaxyHeteromer

The protein–protein complex structure prediction method, which contains GalaxyHeteromer as a new component in addition to GalaxyHomomer (10), participated in the assembly category of CASP14 and CASP14-CAPRI challenges as group name Seok, and they were ranked as fourth and first, respectively (https://predictioncenter.org/casp14/doc/presentations/). The complex structure prediction method shares the same components for both hetero- and homo-oligomer structure prediction in terms of subunit structure prediction, template-based docking, *ab initio* docking, and complex structure refinement.

The performance of GalaxyHeteromer is compared to that of *ab initio* docking method GalaxyTongDock_A (14) on a test set of 143 heterodimers of the Docking benchmark 5 (29), in order to evaluate the combined effect of template-based and *ab initio* docking compared to *ab initio* docking alone. The same monomer models generated by GalaxyHeteromer were used as input subunit structures for *ab initio* docking. To simulate a rather difficult prediction case, the subunit templates with sequence identities >70% were excluded for monomer modelling, and the protein templates with sequence identities of any subunits >70% to the corresponding subunits of the test proteins were excluded for heterodimer modelling. As shown in Table 1, GalaxyHeteromer and GalaxyTongDock_A generate models with better than acceptable quality in CAPRI criterion (30) in 30% and 5% of cases, respectively, as top 1, and in 50% and 34% of cases, respectively, within top 50.

**Table 1.** Performance comparison of GalaxyHeteromer, which combines template-based and *ab initio* docking, with that of GalaxyTongDock_A, which employs *ab initio* docking, in terms of CAPRI criterion of model accuracy on a test set of 143 protein complexes

% of the cases with medium/acceptable quality models within top N		
N	GalaxyHeteromer	GalaxyTongDock_A
**1**	13.3/30.1	1.4/4.9
**5**	18.2/39.2	5.6/13.3
**10**	19.6/41.3	7.0/16.8
**50**	22.4/49.7	9.8/34.3

Next, the performance of GalaxyHeteromer is compared to that of HDOCK (9), which is one of the best available web servers, on the 54 heterodimers used previously for benchmarking HDOCK (9). The protein templates with sequence identities to the target complex greater than 30% were excluded, and unbound subunit structures were used as input. As can be seen from Table 2, GalaxyHeteromer outperformed HDOCK except for the case of top 1 prediction. Top N (*N* = 1, 5, 10 and 50) success rates (percentage of the cases in which models better than acceptable qualities are obtained within the N models) are 33.3%, 53.7%, 55.6% and 68.5%, respectively, for GalaxyHeteromer, whereas those for HDOCK are 38.9%, 40.7%, 44.4% and 59.3%, respectively.

**Table 2.** Performance comparison of GalaxyHeteromer with that of HDOCK in terms of CAPRI criterion on a test set of 54 protein complexes

% of the cases with acceptable quality models within top *N*		
*N*	GalaxyHeteromer	HDOCK
**1**	33.3	38.9
**5**	53.7	40.7
**10**	55.6	44.4
**50**	68.5	59.3

GalaxyHeteromer puts more emphasis on providing multiple alternative solutions for possible complex structures by exploring multiple templates when compared to HDOCK. The provided multiple models may be combined with separate experimental information to select more feasible complex structures.

## THE GALAXYHETEROMER SERVER

### Hardware and software

The server runs on a cluster of 25 Linux servers of 2.40 GHz Intel Xeon E5-2620 v3 12-core processors. The overall GalaxyHeteromer pipeline is implemented using Python. Several components of the pipeline, such as *ab initio* docking and complex refinement, are implemented in the GALAXY programme package written in Fortran90. The web application uses the Python programming language and MySQL database. The JavaScript Protein Viewer (http://biasmv.github.io/pv/) is used for the visualization of models.

### Input and output

#### Input

Amino acid sequences in FASTA format or 3D structures in PDB format for two subunit proteins are the required input. The number of residues in each subunit is restricted as <1000 for computational efficiency. Average run time is 4 h, when structures of both the subunits are provided, and 16 h, when only sequences are provided. It usually takes longer for larger proteins.

#### Output

**Figure 2.**
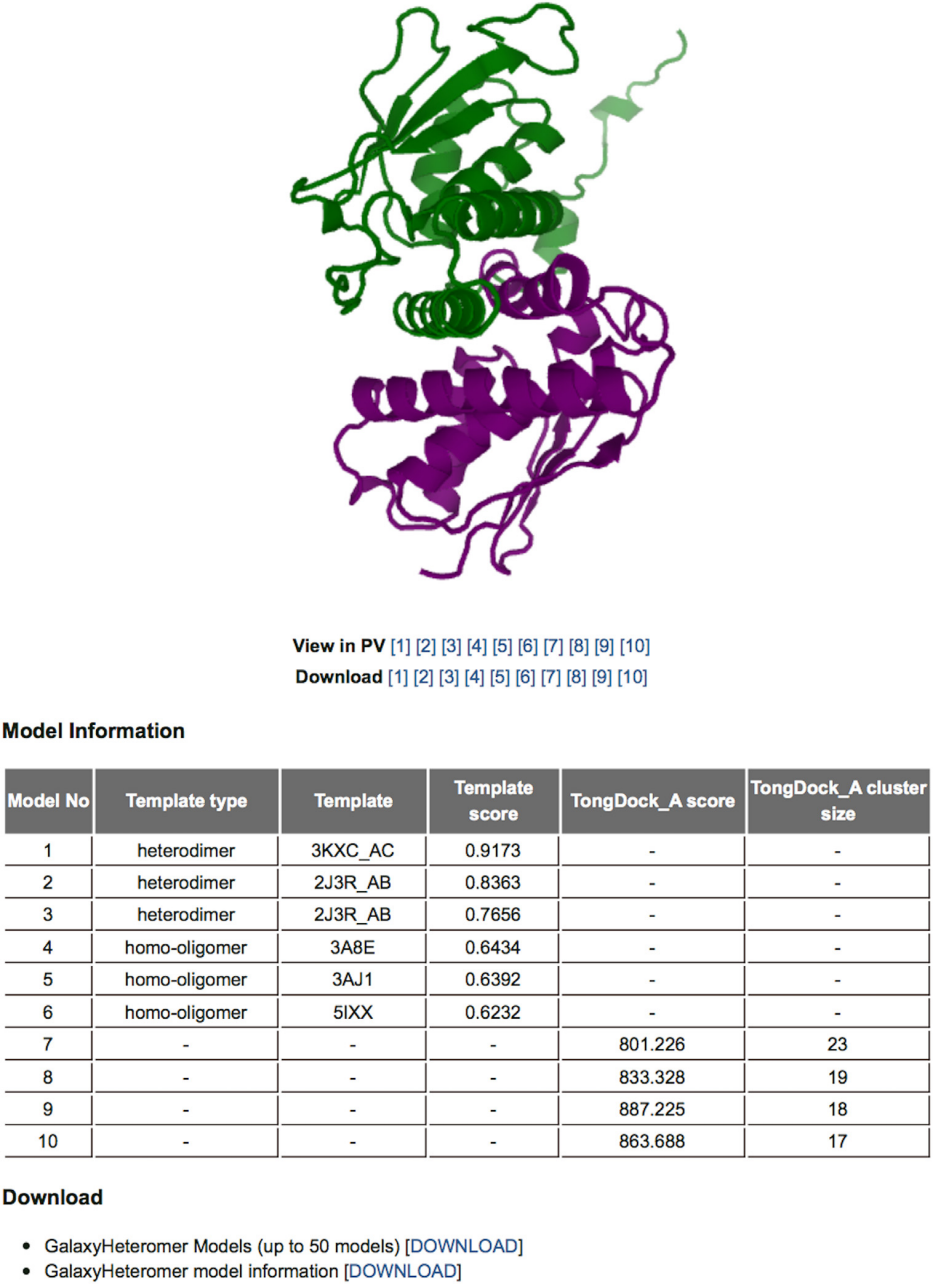
An example output page for GalaxyHeteromer.

On the output page, 10 models are visualized and the following information associated with the models is provided in a table: template type (heterodimer, monomer, or homo-oligomer); template PDB ID and template score for models generated by template-based docking; and GalaxyTongDock_A score and cluster size for models generated by *ab initio* docking. User can also download up to 50 models and information associated with them. An example output page is shown in Figure 2.

## CONCLUSIONS

Herein, a newly developed web server, GalaxyHeteromer, is presented for prediction of heterodimer protein complex structure. The server predicts heterodimer structure from sequences or structures of composing subunits. If subunit structures are unavailable, the server automatically predicts them by up-to-date template- and distance-prediction-based structure prediction methods. GalaxyHeteromer performs both template-based and *ab initio* docking for maximum performance, depending upon the availability of templates in the structure database. In template-based docking, evolutionary relationships of a target protein complex with the domains/subunits of monomer/homo-oligomer proteins, as well as with the subunits of hetero-oligomers, are detected. The provided multiple complex structures may be combined with the available experimental data to select more feasible models for explaining biological functions or designing molecules regulating the functions.

## DATA AVAILABILITY

The GalaxyHeteromer web server is available at https://galaxy.seoklab.org/heteromer. The databases, DB-Mo, DB-Ho, and DB-Het, and the model structures generated for the benchmark sets and per-model accuracy metrics can be downloaded at http://galaxy.seoklab.org/suppl/heteromer.html.

## Notes

Present address: Minkyung Baek, Department of Biochemistry, University of Washington, Seattle, WA 98195-1655, USA.
